# Is hyaluronan deposition in the stroma of pancreatic ductal adenocarcinoma of prognostic significance?

**DOI:** 10.1371/journal.pone.0178703

**Published:** 2017-06-05

**Authors:** Florian Gebauer, Marius Kemper, Guido Sauter, Peter Prehm, Udo Schumacher

**Affiliations:** 1 Department of General, Visceral and Thoracic Surgery, University Medical Centre Hamburg-Eppendorf, Hamburg, Germany; 2 Centre of Experimental Medicine, Department of Anatomy and Experimental Morphology, University Medical Centre Hamburg-Eppendorf, Hamburg, Germany; 3 Centre for Diagnostic, Department of Pathology, University Medical Centre Hamburg-Eppendorf, Hamburg, Germany; 4 Hylitis, Nottuln, Germany; University of Nebraska Medical Center, UNITED STATES

## Abstract

**Background:**

Pancreatic ductal adenocarcinoma (PDAC) has a dismal prognosis and the number of PDAC-related deaths is rising. Recently the tumour stroma and in particular one of its main components, hyaluronan (HA), have attracted considerable attention as intravenous hyaluronidase treatment together with conventional chemotherapy considerably prolonged survival in HA-rich PDA patients. We therefore wanted to investigate the prognostic significance of HA deposition in PDA using both antibodies to HA and hyaluronan binding protein (HABP).

**Material and methods:**

Tissue microarrays of PDAs of 184 patients and pancreatic xenografts tumours were immunohistochemically (IHC) stained for HA using either biotinylated hyaluronic acid binding protein (HABP) or anti-HA antibody.

**Results:**

The pattern of staining with HABP differed significantly from that with antibody IHC. Antibody staining was found both within cancer cells and in the extracellular matrix and staining could not be eliminated by hyaluronidase predigestion of the tissue sections. In contrast, HABP staining was generally confined to the extracellular matrix and was completely abolished by hyaluronidase pretreatment. HA positivity as determined by HABP was associated with larger primary tumours (p = 0.046). There were no correlations between overall survival, disease-free survival and HA expression.

**Conclusion:**

Presence of HA alone is not of prognostic importance in PDAC, and IHC with utilization of antibody detection shows no reliable staining pattern and should not be applied for HA IHC.

## Introduction

Despite intensive research efforts, the prognosis of patients suffering from pancreatic ductal adenocarcinoma (PDA) has not improved during the last few decades [1]. Therefore novel therapeutic approaches are urgently required to improve the clinical situation. One such approach might be modulation of the extracellular matrix (ECM) of carcinomas in order to enhance cancer therapy [2, 3]. One particular component of the ECM, namely hyaluronan (HA), has attracted considerable interest. It is a large polyanion of the ECM that allows extensive hydration, thus occupying a large hydrodynamic volume [4]. By combining pegylated hyaluronidase to degrade HA in the ECM with gemcitabine, a considerable therapeutic benefit was observed in a clinical study by Hingorani et al. in a small number of PDA patients whose tumour stroma was rich in HA [5]. The observation that a HA-rich stroma is associated with significantly shorter survival had already been made by Cheng and colleagues [6]. These two studies used different methodologies to detect HA: Hingorani used hyaluronan binding protein (HABP) to detect HA in tissue sections [5] while Cheng and colleagues used antibodies to detect HA [6]. As shown for carbohydrate histochemistry in particular, differences in the methods used can severely influence the results of survival analyses, hence we also evaluated the influence of the two methods on the results of HA detection in tissue sections.

## Material and methods

### Study design and patients

This study was approved by the ethics committee of the chamber of physicians, Hamburg, Germany. Written informed consent was obtained from all patients to use the resected tumour samples. For this study, 264 patients with PAC who underwent surgery at the University Medical Centre Hamburg-Eppendorf between February 1994 and May 2005 were analysed. None of the patients received neoadjuvant treatment. All data including sex, histology, tumour size, lymph node metastasis and disease stage (UICC 6^th^ edition) were obtained from review of a combination of clinical and pathological records, from outpatient clinic medical records and communication with patients and their attending physicians.

### Tissue microarray (TMA) construction and analysis

Tissue cores were obtained from pathologically proven formalin-fixed paraffin-embedded (FFPE) tissue blocks of PAC. Based on haematoxylin-eosin staining representative areas of the tumour were selected.

TMA construction was performed as described previously [7]. Briefly, 358 tissue cylinders with a diameter of 0.6 mm were punched from the ‘‘donor” tissue blocks using a homemade semiautomatic robotic precision instrument and brought into one recipient paraffin block containing 358 individual samples. Among these samples there were 264 PDAC, 33 intraductal papillary mucinous neoplasms (IPMN), 40 neuroendocrine pancreatic tumours (NET) and 36 samples of healthy tissue as negative controls. Four-micrometre sections of the resulting TMA blocks were transferred to an adhesive-coated slide system (Instrumedics Inc., Hackensack, New Jersey). The staining pattern was analysed using a modified analysis protocol as previously described [8]. In brief, the staining intensity (0, 1+, 2+, 3+) was scored for each tissue spot. Spots without staining and with a staining intensity of 1+ of the extracellular stroma were scored as HA negative; positive scores were given for a staining intensity of 2+ and more. Staining of intracellular compartments was not considered for analysis. Immunohistochemical analysis of the sections was performed without knowledge of the patients’ identity or clinical status.

### Hyaluronan detection in tissue sections

Both human nasal conchae previously used as control tissue for HABP [9] and primary xenografts of a human pancreatic adenocarcinoma cell line (PaCa 5061) [10, 11] were used as control tissues for the detection of HA and for the hyaluronidase digestion experiments.

### Ligand binding histochemistry using hyaluronic acid binding protein (HABP)

Sections were deparaffinized and dried overnight at 37°C. Sections were incubated in a water bath at 60° C in a 1:10 diluted DAKO retrieval solution (# S1699, Dako, Glostrup, Danmark). After cooling down for 30 minutes half of the retrieval solution was replaced by distilled water. After 10 minutes sections were placed in distilled water for 5 minutes. After two washes in Tris-buffered saline (TBS; 0.05 M Tris-HCl at pH 7.6 and 0.15 M NaCl) for 5 minutes, the sections were incubated with 1% bovine serum albumin in TBS for 30 minutes (Dako). Biotinylated hyaluronic acid binding protein (HABP, # 385911 Calbiochem, Merck, Darmstadt, Germany) diluted 1:75 in Antibody Diluent (# B 1-31C, Medac, Wedel, Germany) was applied for 1 h at room temperature. The binding sites were detected using the ABC-AP-Kit (Vector Laboratories Inc., Burlingame, CA). The Permanent Red Kit (Dako) was used as a chromogen. Sections were counterstained with hemalumn (Merck, Darmstadt, Germany), dehydrated and covered with Eukitt (Kindler, Freiburg, Germany).

### Immunohistochemical detection of hyaluronan using a polyclonal antibody

For antigen retrieval, deparaffinized sections were microwaved three times for 5 minutes each in a pH 6 citrate buffer at the maximum setting of the microwave oven. Afterwards the sections were treated in the same buffer for 20 minutes in a water bath at 90°C. After cooling down to room temperature, sections were washed twice in TBS for 5 minutes each and sheep anti-HA polyclonal antibody (Abcam ab 53842, Cambridge, UK) diluted 1: 200 in Antibody Diluent (S3022, Dako) was applied at 4°C overnight. After washing twice in TBS plus 0.2% Triton X 100 there was a further wash for 5 minutes in TBS. A biotinylated rabbit anti-goat secondary antibody (Dako) diluted 1: 200 in TBS was applied for 30 minutes. Normal rabbit control serum (# X0903, Dako) was used as a negative control. For the detection of the antigen binding sites the same ABC-AP-Kit and visualization procedures as described above were used.

### Specificity control using hyaluronidase digestion

*Streptomyces* hyaluronidase (389561-100U, Merck) was dissolved in 1 ml 0.02 M sodium acetate buffer pH 6.0. This stock solution was diluted 1:10 in the sodium acetate buffer for final use. Deparaffinized sections were treated three times for 5 minutes each in the sodium acetate buffer and thereafter for 4 hours at 37°C in 10 U/ml hyaluronidase in acetate buffer. This was followed by a fresh hyaluronidase 10 U/ml solution for another 20 hours. After two washes in TBS plus Tween 20 and one additional wash in TBS alone, the primary antibody was applied.

### Statistical analysis

SPSS Statistics for Mac OS (Version 20, SPSS Inc., Chicago, IL USA) was used for statistical analysis. Relationships between the immunostaining and clinico-pathological data were calculated using Chi-square and Fisher’s Exact tests. Survival curves were plotted using the Kaplan-Meier method and analysed by log-rank test. Multivariate analysis was performed using the Cox regression model. All tests were two-sided. P-values less than 0.05 were considered statistically significant.

## Results

### HA deposition in PaCa 5061 xenograft primary tumours

**Fig 1:**
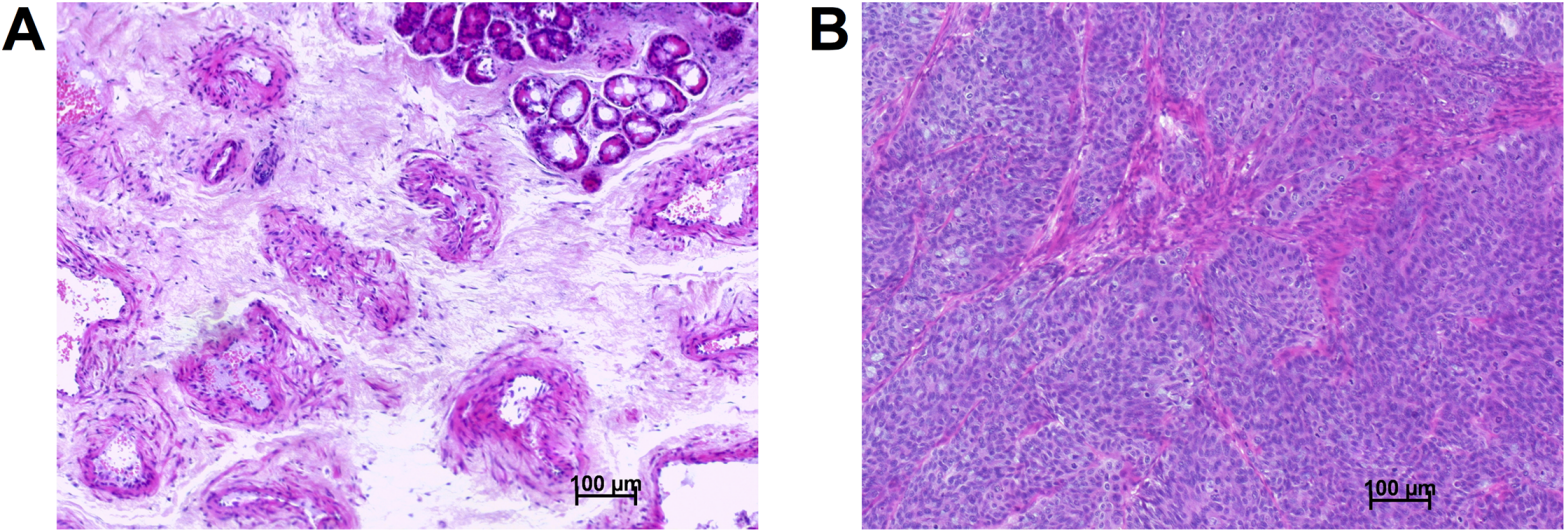
Haematoxylin and eosin (H&E) staining of human nasal conchae (A) and PaCa 5061 xenograft tumour (B).

**Fig 2:**
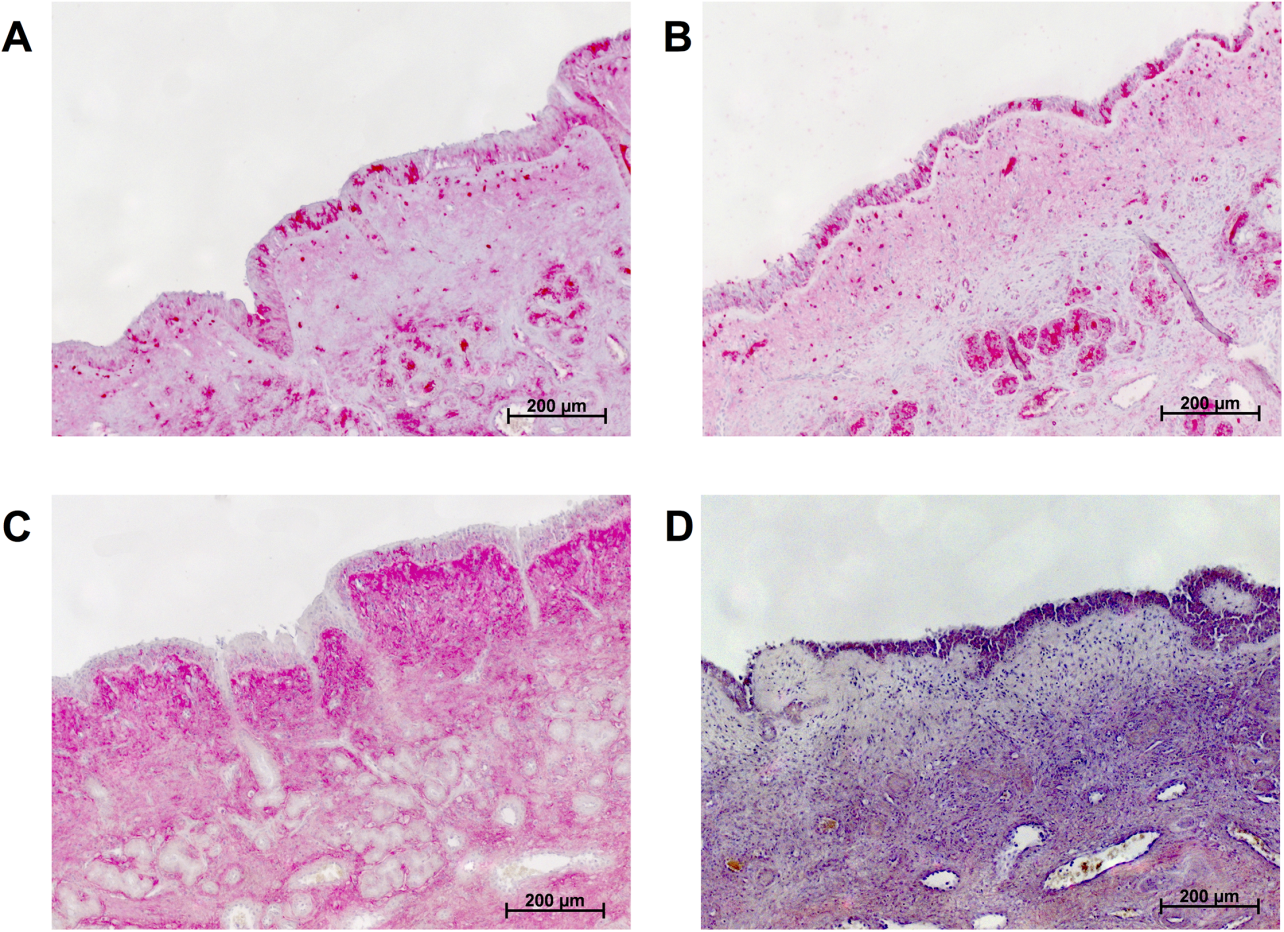
Hyaluronan (HA) deposition in human nasal conchae as determined by anti-HA antibody staining (A) and after hyaluronidase pretreatment (B). Changes in staining patterns and intensity were not seen, unlike when detecting HA by hyaluronic acid binding protein (HABP) ligand histochemistry (C), which showed a complete absence of HA after pretreatment (D).

**Fig 3:**
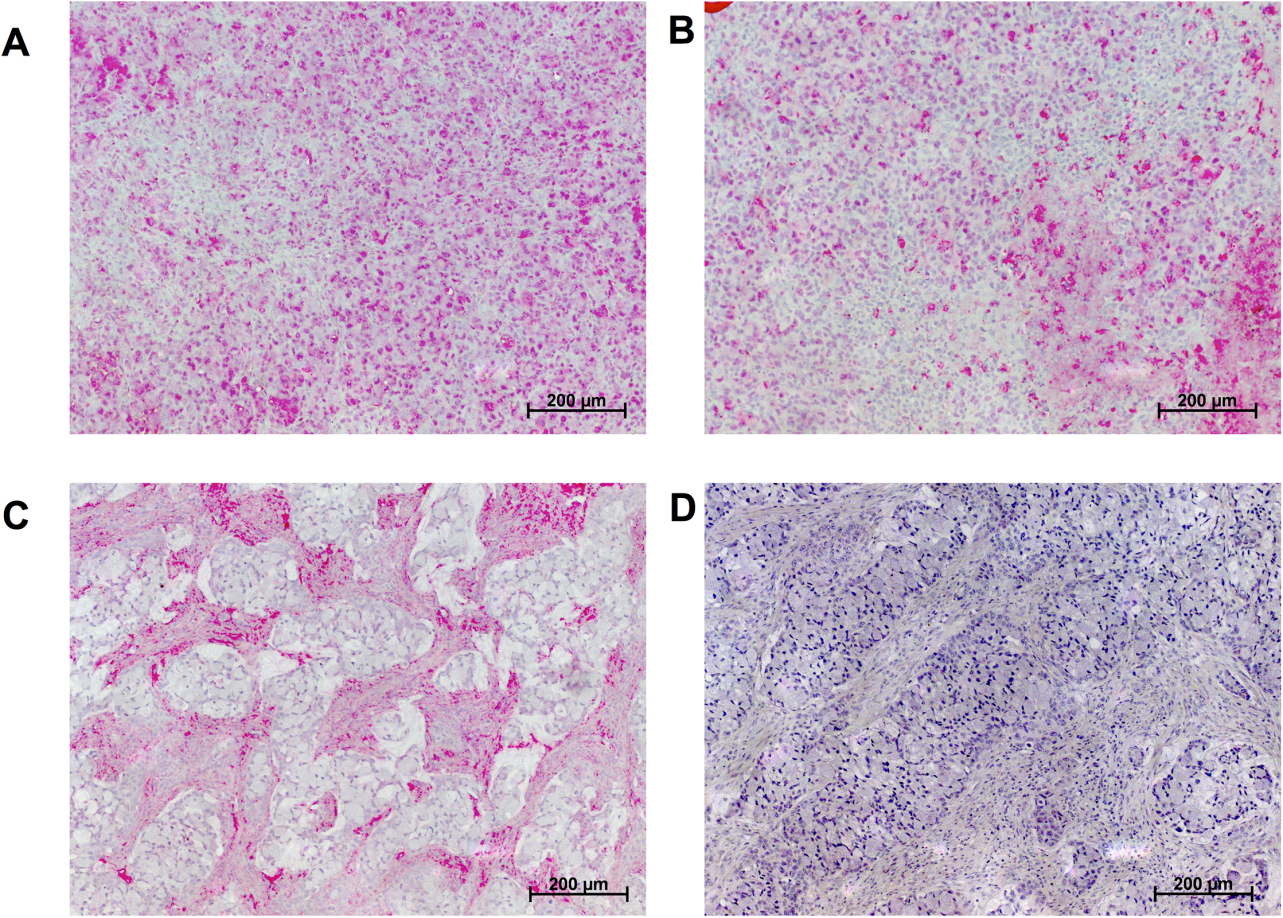
PaCa 5061 primary human pancreatic adenocarcinoma xenograft immunohistochemically stained with an anti-hyaluronan polyclonal antibody. Note the staining of the cancer cells themselves, which were more intensively stained than the surrounding extracellular matrix (A). If hyaluronan was detected by hyaluronic acid binding protein, it was present in the extracellular matrix and not in the cancer cells themselves (C). If the section was pre-treated with hyaluronidase, the hyaluronan immunoreactivity did not vanish after antibody IHC (B); in contrast, hyaluronidase pre-treatment abolished its reactivity completely according to HABP staining (D). Collectively these results indicate that the anti-hyaluronan antibody binding is not caused by hyaluronan and that this approach is therefore unsuitable for its detection.

To validate the staining method, human nasal conchae were used as a positive control as high basal HA expression has been described previously in this tissue (H&E staining depicted in Fig 1) [8]. Immunohistochemistry performed using a polyclonal antibody showed non-specific binding as it was abolished after hyaluronidase pretreatment (Fig 2). Similar results were found for xenografts tumours of a human cancer cell line (Fig 3). Using the polyclonal antibody against HA, moderate HA immunolabelling was noted in the cancer cells while weak or no labelling was noted in the extracellular matrix. Again, the labelling intensity was not diminished by hyaluronidase pre-treatment. In contrast, HABP stained the extracellular matrix only and not the cancer cells and all HABP binding was completely abolished by hyaluronidase pre-treatment.

### TMA results

HA expression was evaluable in 184 cases. Reasons for non-informative cases (n = 80; 30%) included complete lack of tissue samples or absence of unequivocal cancer tissue in the TMA section. Five patients (2.7%) died within 30 days after surgery due to surgically related or perioperative complications and were not considered for the analysis. Finally, 175 patients were included in the survival analysis.

Characteristics of patients with interpretable tumours are listed in Table 1. Briefly, the median age of the study population was 63.4 years (range 32–87.5 years), 103 were male (56.0%), 81 female (44.0%). All patients underwent pancreaticoduodenectomy between 2000 and 2012, none was treated with neoadjuvant therapy, while 162 (88.8%) patients received adjuvant gemcitabine chemotherapy, and none underwent postoperative radiotherapy. One hundred and forty patients received a R0 resection (72.2%), and 44 (27.8%) patients a R1 resection.

**Table 1:** Clinico-pathological data of patients with pancreatic adenocarcinoma correlated with hyaluronan (HA) expression levels (*n*.*a*. not available).

			HA expression		
		total	negative	positive	*p* value
All patients		184	22	162	n.a.
			13.6%	86.4%	
Sex	male	103	14	89	0.296
			13.6%	86.4%	
	female	81	8	73	
			9.9%	90.1%	
Age group	< 65 years	99	13	86	0.383
			13.1%	86.9%	
	> 65 years	85	9	76	
			10.6%	89.4%	
Tumour stage	pT1	5	2	3	0.046
			40.0%	60.0%	
	pT2	47	2	45	
			4.3%	95.7%	
	pT3	121	17	104	
			14.0%	86.0%	
	pT4	7	0	7	
			0.0%	100.0%	
Lymph node stage	pN0	63	9	54	0.632
			14.3%	85.7%	
	pN1	116	12	104	
			10.3%	89.7%	
Distant metastasis	M0	177	21	156	0.596
			11.9%	88.1%	
	M1	7	1	6	
			14.3%	85.7%	
Grading	G1	8	1	7	0.618
			12.5%	87.5%	
	G2	82	12	70	
			14.6%	85.4%	
	G3	92	9	83	
			9.8%	90.2%	
Resection status	R0	140	25	115	0.409
			18.2%	81.8%	
	R1	44	5	39	
			11.8%	88.2%	

**Fig 4:**
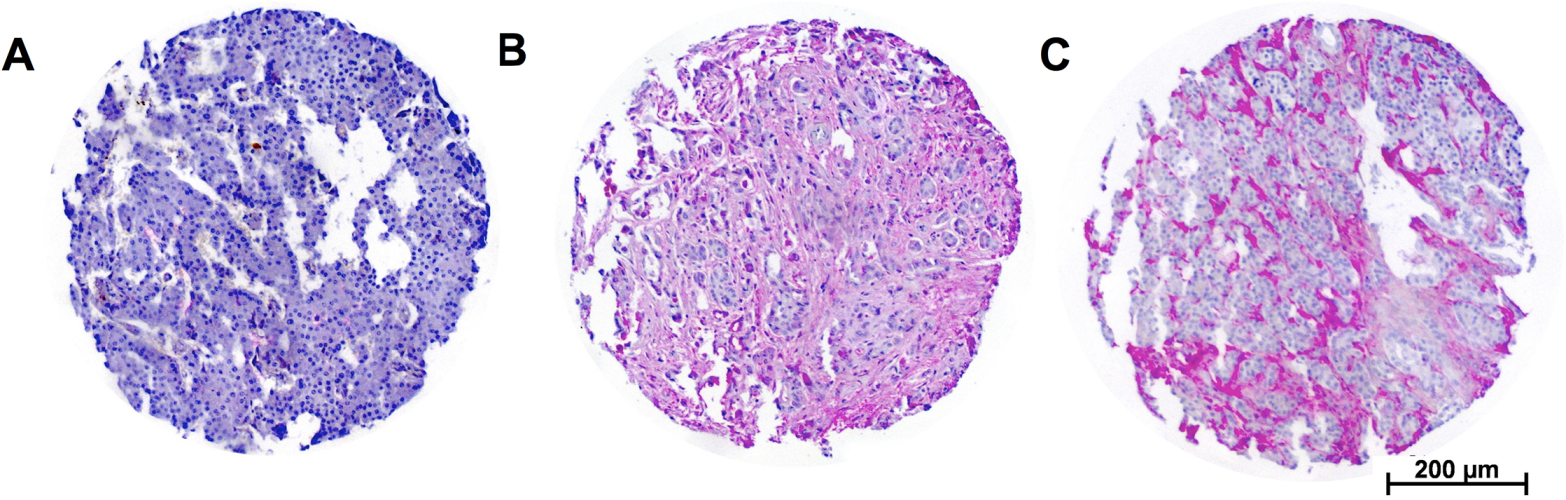
Hyaluronan deposition in pancreatic ductal adenocarcinoma as determined by hyaluronic acid binding protein (HABP) ligand histochemistry. Some tumours showed no extracellular HA (A) deposition while other tumours showed moderate (B) or high amounts of HA (C).

HA was expressed in 162 patients (86.4%), while 22 patients showed no HA expression (13.6%). HA showed an extracellular distribution within the tumour and no intracellular accumulation of HA within tumour cells was noted (Fig 4). Staining was not seen on normal acinar and ductal cells of the peritumoural areas. A correlation between HA staining and the primary tumour size was observed. Larger tumours showed HA expression significantly more often than smaller ones did (p = 0.046). Apart from that, there was no correlation with other clinic-pathological data (Table 1).

### Overall and disease-free survival

**Fig 5:**
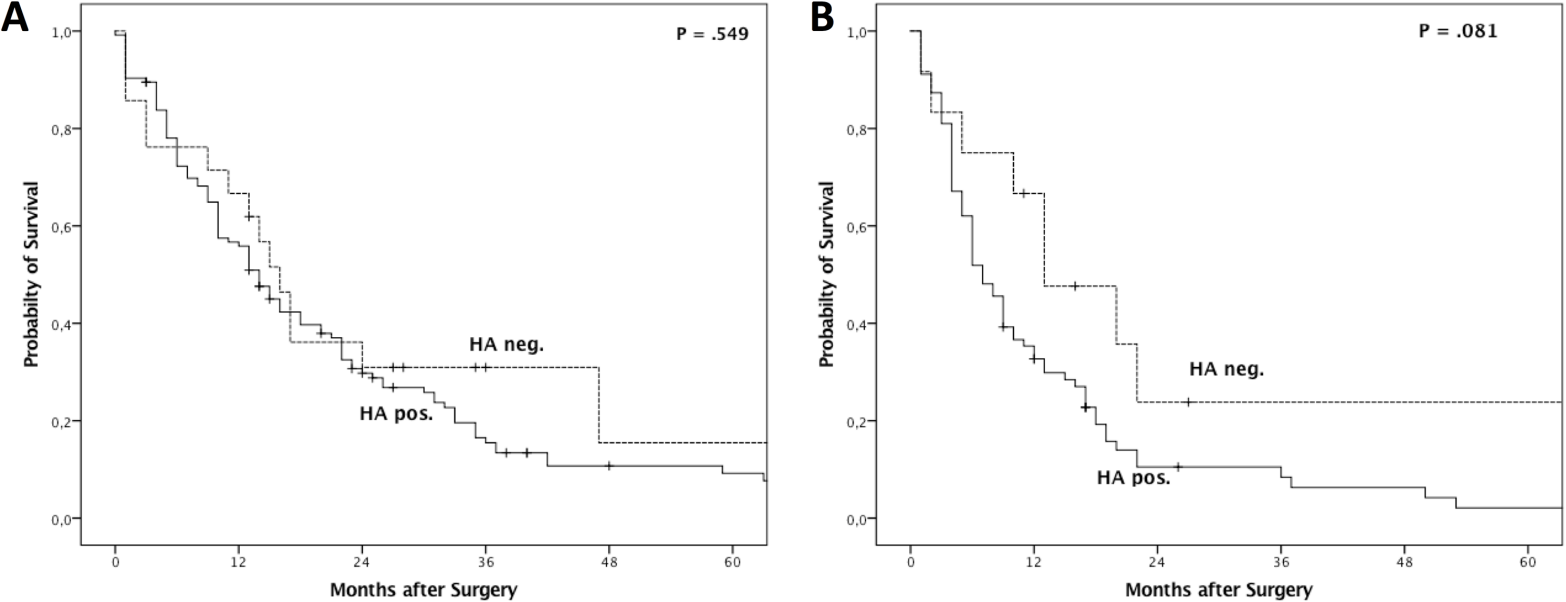
Kaplan-Meier survival analysis for overall survival (A) and disease-free survival (B) in patients with PDAC after surgical tumour resection.

Median overall survival for all patients was 14.0 months (95%CI 11.7–16.3 months), and median disease-free survival was 8.0 months (95%CI 5.9–10.1 months), respectively. Median overall survival was 16.0 months (95%CI 12.9–19.2 months) in patients with positive HA expression, and 14.0 months (95CI 11.5–16.5 months, P = .549) in patients without HA expression (Fig 5A). Disease-free survival in patients with and without HA expression showed a similar pattern, with a slightly shorter disease-free survival (7.0 months, 95%CI 5.1–8.9 months) in patients with HA expression versus those without (13.0 months, 95%CI 3.5–22.5 months, P = 0.081) (Fig 5B). Survival analysis was also performed with R0 resected patients only. Overall and disease-free survival did not show any significant differences in terms of correlation with HA expression status (data not shown).

## Discussion

The present study was undertaken to evaluate the possible prognostic role of HA deposition in the extracellular matrix of pancreatic ductal adenocarcinoma and identify patient subgroups with high HA expression profiles that may profit from hyaluronidase treatment. In our series we were unable to find a prognostic impact of HA deposition on the patients´ survival although the vast majority of patients (88%) showed at least moderate or high expression of HA in the extracellular matrix. In addition, larger tumours were associated with higher levels of HA expression.

Pancreatic cancer is characterized typically by a dense desmoplastic stroma containing a large amount of extracellular matrix such as collagen, fibronectin, laminin and HA [12, 13]. The biological functions of a HA-rich microenvironment in malignant tumours have been investigated in preclinical trials and showed that presence of HA may promote tumour progression by enhancing cell proliferation, migration, invasion, neo-angiogenesis and increased resistance to chemo- or targeted cancer therapy [12, 14, 15]. Increased levels of HA were already correlated with increased tumour progression and shortened overall-survival in breast cancer [16], colorectal cancer [17], gastric cancer [18] and prostate cancer [19]. Animal models revealed accelerated tumour growth following HA accumulation by overexpression of HA-synthase [20]. These findings suggest that HA is a potential target for improved cancer-specific therapy in PDAC. A recently published phase Ib trial by Hingorani and colleagues analysed the safety and tolerability of PEG-hyaloronidase in patients with PDAC in combination with gemcitabine, with promising results for future clinical trials [5]. The results of our study underline the importance of HA in PDAC, despite the lack of significant differences in OS in our series. However, the fact that almost all tumours show high HA expression underlines the biological importance of HA in tumour progression. The fact that HA expression was seen in large tumours more often than in small tumours is consistent with the aforementioned observations as the synthesis of large amounts of ECM is obviously mandatory during tumour progression in PDAC [3, 21, 22].

The results of our present study are contrast with those of a recent study published by Cheng and colleagues who were able to show a strong correlation between HA expression and patients’ overall survival although larger tumours were correlated with higher HA expression in our series [6]. However, different methodologies were used in our study. Cheng and colleagues used a polyclonal antibody against hyaluronan, which resulted in strong staining of the pancreatic cancer cells (Fig 2). As hyaluronan is a non-immunogenic polysaccharide [23] we found this result astonishing and used this antibody in parallel with our established HABP histochemistry [9]. This staining method had been validated previously and also used in recent clinical studies for analysis of baseline HA expression. Indeed, the antibody used by Cheng et al. [6] was also used by us and showed the same staining pattern in our pancreatic tumours, namely the staining was present in the cancer cells themselves. However, this staining could not be abolished by hyaluronidase predigestion. This is in contrast to the HABP binding sites, which were present in the extracellular matrix and could be abolished completely by hyaluronidase predigestion. We therefore assume that the presence of HA cannot adequately be determined by using this antibody. Hence no conclusion with respect to prognosis and HA deposition can be drawn from this paper because of the methodological problems.

According to our findings, HA in itself is not of prognostic relevance. As almost 90% of the tumours were HA positive, the expression of HA is likely to be of great importance during tumour progression in PDAC, as illustrated by the extensive desmoplastic reaction in almost every PDAC. Interestingly, our results revealed higher levels of HA expression in larger tumours and 100% of pT3/4 tumours were HA positive. Obviously HA expression becomes mandatory at some point during tumour progression in order to provide an optimized tumour microenvironment for further tumour growth. Unfortunately, we were not able to analyse PanIN precursor lesions with respect to HA expression but it would be interesting if early HA expression could be correlated with later transformation in malignant tumours.

As immunohistochemistry using antibodies to HA has proven to be unreliable, the stratification of patients according to high and low HA content should therefore be made by HABP histochemistry. The increased survival of pancreatic cancer patients treated by pegylated hyaluronidase and gemcitabine [5] is therefore due to a drug-mediated effect and not caused by differences in HA deposition pattern, as this pattern is not indicative of prognosis in pancreatic adenocarcinoma patients.
